# Biochemical Profile by GC–MS of Fungal Biomass Produced from the Ascospores of *Tirmania nivea* as a Natural Renewable Resource

**DOI:** 10.3390/jof7121083

**Published:** 2021-12-17

**Authors:** Jamal M. Khaled, Naiyf S. Alharbi, Ramzi A. Mothana, Shine Kadaikunnan, Ahmed S. Alobaidi

**Affiliations:** 1 Department of Botany and Microbiology, College of Science, King Saud University, Riyadh 11451, Saudi Arabia; nalharbi1@ksu.edu.sa (N.S.A.); sshine@ksu.edu.sa (S.K.); ahalobaidi@ksu.edu.sa (A.S.A.); 2 Department of Pharmacognosy, College of Pharmacy, King Saud University, Riyadh 11451, Saudi Arabia; rmothana@ksu.edu.sa

**Keywords:** GC–MS analysis, *Tirmania nivea*, antimicrobial, anticancer, production

## Abstract

The edible fruiting bodies of desert truffles are seasonally collected and consumed in many regions of the world. Although they are very expensive, they are bought and sold as a result of considerable scientific reports confirming their health and nutritional benefits. This study aimed to conduct laboratory production of the fungal biomass of *Tirmania nivea* as a natural renewable resource of many active biological compounds using an artificial growth medium. The *T. nivea* collected from Hafar Al-Batin, which is north of Saudi Arabia, and their ascospores were harvested and used to produce fungal biomass in potato dextrose broth. The cultivation was conducted using a shaking incubator at 25 °C for two weeks at 200 rpm. The crud extracts of the fungal biomass and mycelium-free broth were prepared using ethyl acetate, methanol and hexane. Preliminary gas chromatography–mass spectrometry (GC–MS) analysis and their biological activity as antimicrobial agents were investigated. The results showed that the crude extracts have biological activity against mold, yeast and bacteria. The preliminary GC–MS analysis reported that the fungal biomass and extracellular metabolites in the growth medium are industrial renewable resources of several biological compounds that could be used as antifungal, antibacterial, antiviral, anticancer, antioxidant, anti-trypanosomal and anti-inflammatory agents.

## 1. Introduction

*Tirmania nivea*, which is locally called zubaidi, fagaa or kma’at, is an edible hypogenous fungal fruiting body. From a taxonomic point of view, the genus *Tirmania* belongs to the family Terfeziaceae and the order Pezizales. *T. nivea* is collected seasonally from several desert environments, including Saudi Arabia’s desert. It is consumed because of its distinctive flavor and nutrient value, and it is relatively expensive due to its natural and seasonal growth associated with specific conditions and limited environments [1,2].

Generally, desert edible fungal fruiting bodies are ethnopharmacologically used to treat eye infections and fatigue, as well as promote fertility in men [3]. Chemical analysis showed that 100 g of a dry fruiting body of *T. nivea* consisted of fat, protein, carbohydrates and ash at 6.78, 28.8., 57.8 and 5/100 g, respectively; ascorbic acid, carotenoids and anthocyanins at 10.6, 1.1 and 29.1/100 g; and minerals such as potassium, calcium, magnesium, iron, sodium, phosphate, manganese and copper [4]. 

The major volatile organic compounds of the ascocarp of *T. nivea* include unsaturated fatty acids and hexa-decanoic acid [5]. Antioxidant chemicals extracted from the fruiting body of *T*. *nivea* vary according to its natural source. Generally, it contains anthocyanins, ascorbic acid, phenolics, flavonoids and carotenoids [6]. Aside from using truffles as food, many studies have proven that they are an important source of biological compounds, such as antioxidant, antibacterial, antifungal, antiviral, hepato-preservative, anticancer and anti-inflammatory agents [7]. A crude ethyl acetate extract obtained from *T. pinoyi* showed biological activity as an antimicrobial agent against *Enterococcus* sp., *Staphylococcus aureus*, *Bacillus subtilis*, *Pseudomonas aeruginosa* and *Escherichia coli* [8]. Some alcoholic extracts produced from the ascocarp of *T. nivea* and *Terfezia claveryi* showed biological activity against the human liver cancer cell line HepG2 [9]. The aqueous extracts of some desert truffles could be used to produce nanoparticles, which could be introduced in some applications [10].

Ultra-high performance liquid chromatography coupled with mass spectrometry (UPLC-MS) is used to analyze fruiting bodies of *T. nivea* and *T. claveryi*. It was reported that *T. nivea* and *T. claveryi* fruiting bodies contain behenic acid, resveratrol, margaric acid, naringenin, oleic acid, and lauric acid and that the highest hit bio-compounds related to anti-inflammatory targets [11]. The fruiting bodies of these truffles have a distinctive aroma because they contain significant volatile bio-compounds such as esters, alcohols, aldehydes, ketones, and sulfurs. Their therapeutic features involve their biological activity as antioxidants, antiviral, antimicrobial, hepatoprotective, and inflammatory agents. The major bio-compounds responsible for curative properties include phytosterols, N-arachidonoyl-ethanolamine (anandamide), phenolics, and steroidal glycosides (tuberoside) [12]. 

Although there have been successful attempts to cultivate some types of desert truffle, such as *T. claveryi* [13], there are many challenges related to climatic conditions and weather effects. Given the high yields of mycelia that can be produced in a laboratory, this work was designed to produce mycelia of *T. nivea* using artificial media in the laboratory and to perform a gas chromatography–mass spectrometry (GC–MS) analysis of several alcoholic extracts to determine the active biological compounds from the produced mycelia. 

## 2. Methodology

### 2.1. Sample Collection

Edible desert truffles (*T. nivea*) were collected from Hafar Al-Bati, which is north of Saudi Arabia (coordinates: 28°26′3″ N 45°57′49″ E), between 1 March and 30 April 2021. The samples were immediately transferred using iceboxes to the Microbiology Laboratory, Botany and Microbiology Department, College of Science, King Saud University, Riyadh, Saudi Arabia. The morphological features of the samples were recorded, and the samples were washed three times using water. The external surface of the samples was sterilized three times using an ethanol solution (70% ethanol), and then the samples were washed three times using sterile water. The samples were put in sterile plastic boxes and preserved at −20 °C until further analysis.

### 2.2. Macroscopic and Microscopic Study

Morphological characteristics of sporocarp (fruiting body) and ascospore were recorded. A thin layer of the internal part of the samples was examined using digital light microscopy (Motic plus 2 ML, Xiamen, China) without using any stains and with lactophenol cotton blue stain (Sigma-Aldrich, St. Louis, MO, USA). The characteristics of the asci and ascospores were recorded. Colony characteristics grown from the ascospores were studied on potato dextrose agar (PDA) (Oxoid Ltd., Basingstoke, UK) after incubation at 25 °C for 48 h. then the fungal hyphae were examined using light microscopy.

### 2.3. Mycelia Production

The spore suspension was formulated from the internal part of the sample prepared, as mentioned above. The internal part of the sample was vigorously crushed, and centrifugation (Universal 320, Hettich, Zentrifugen, Germany) was conducted at 3000× *g* for 10 min. The supernatant was collected, and a direct microscopic count using Petroff-Hausser counting chambers and a standard plate count using the pour plate method were used to count and determine the viability of the ascospores. The mycelia of *T. nivea* were produced from the ascospore suspension using potato dextrose broth (Oxoid Ltd., Basingstoke, UK) (0.4 g of potato extract produced from 20 g of infused potato and 2 g of dextrose per 100 mL of distilled water) at 25 °C for two weeks at 200 rpm (IKA^®^ KS 4000 i control, Staufen, Germany). Mycelia were collected from the broth using centrifugation at 2000× *g* for 10 min. The collected mycelia were washed three times using sterile normal saline solution (0.89% sodium chloride). The wet biomass was collected, and the yield (g of wet biomass per 100 mL of the medium used for cultivating the mycelium of *T. nivea*) was calculated.

### 2.4. Alcoholic Extracts

The production of crude alcoholic extracts was performed using methanol (Sigma-Aldrich, USA), ethyl acetate and hexane (Avonchem, Macclesfield, UK), and n-hexane (Sigma-Aldrich, St. Louis, MO, USA). Exactly 100 mL of the mycelium-free broth or 10 g of wet mycelial biomass was extracted by 100 mL of solvent through the maceration method at room temperature using a shaking incubator at 200 rpm (IKA^®^ KS 4000 i control, Staufen, Germany). The extraction was repeated three times, and a fresh solvent was used each time. The crude extracts were filtered, and the solvent was removed using a rotary evaporator (IKA^®^ Rv 10, Staufen, Germany).

### 2.5. GC–MS Analysis

Agilent GC 7890A jointed with a triple-axis detector 5975 C single quadrupole mass spectrometer were used for GC-MS analysis. The column of chromatographic was an Agilent HP 5MS column (30 m × 0.25 mm × 0.25 µm film thickness), with gas carrier (high-purity helium, at a flow rate of 1 mL/min). The temperature of the injector was 280 °C and it was equipped with a splitless injector at 20:1. The temperatures were set at 230 °C and 150 °C for the source temperature of MS and the Quad temperature, respectively. The initial oven temperature was 40 °C for 1 min, then it was increased to 150 °C at 10 °C min^−1^ for 1 min, increasing further to 300 °C at 10 °C min^−1^ for 1 min. The scanning range was set at 40 to 600 mass ranges at 70 eV electron energy with solvent holdback of 3 min. Eventually, unknown compounds were identified by comparing the spectra with those of the NIST 2008 (National Institute of Standard and Technology library). The single sample analysis was 29 min as required total time for analysis.

### 2.6. Biological Activity of Crude Alcoholic Extracts

The preliminary biological activity of the crude extracts against bacteria, mold and yeast was tested using a disk diffusion assay. A 6-mm sterile disk was loaded with 4 mg of the crude extract, and the test protocol was followed according to the studies of Desbois and Smith [14] and Khadka et al. [15] using *E. coli* ATCC 25922, *S. aureus* ATCC 29213, *Candida albicans* ATCC 60193 and *Aspergillus niger* Wild strain.

## 3. Results

### 3.1. Macroscopic and Microscopic Characteristics

**Figure 1:**
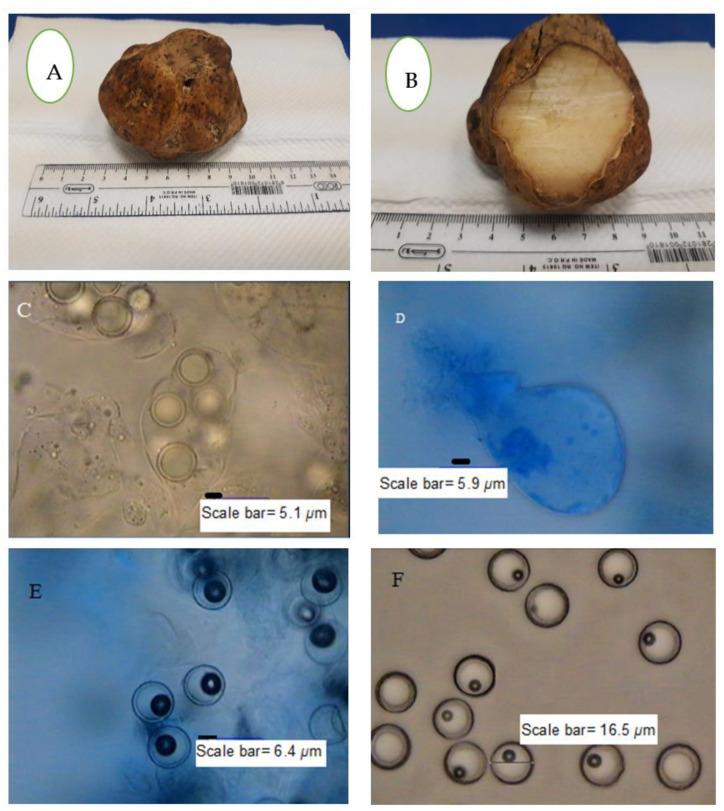
Macroscopic and microscopic features of *T. nivea*. (**A**) Whole fruiting body, (**B**) sporocarp (fruiting body) internal view, (**C**) ascus with aeciospores, (**D**) ascospore-free ascus stained with lactophenol cotton blue, (**E**) free ascospores strained with lactophenol cotton blue and (**F**) free ascospores without stain.

**Figure 2:**
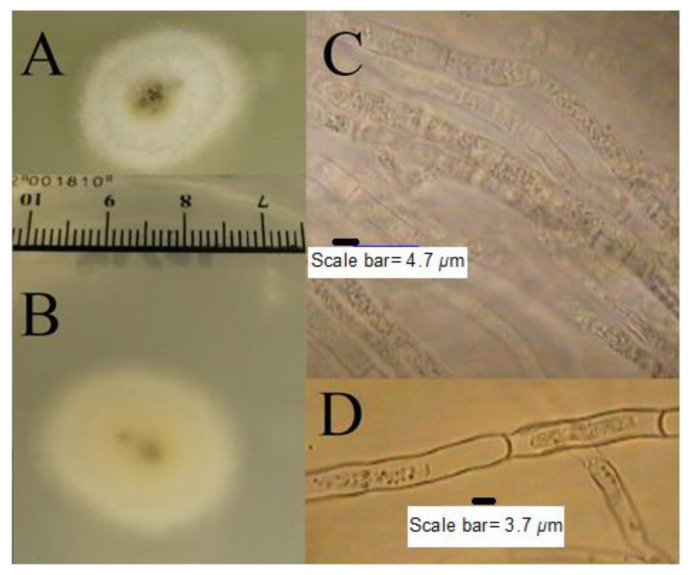
Morphological culture and microscopic features of colony produced from *T. nivea* ascospores on PDA after incubation 48 h at 25 ± 1 °C. (**A**) Front view of the colony, (**B**) reverse view of the colony, and (**C**,**D**), microscopic view of septate-hyphae without staining.

*T. nivea* has a white edible fruiting body with a size that can sometimes reach more than 10 cm. The white color of the fruiting body changes to dark yellow over time as a result of exposure to air. The amyloid ascus of *T. nivea* has an ellipsoid shape, with eight smooth ascospores (Figure 1). Figure 2 shows the morphological culture and microscopic characteristics of colony produced from *T. nivea* ascospores on PDA. Hairy white colonies (2.5 cm in size) after incubation for 48 h, with a light black center and light yellow pigment, can be shown in the reverse view of the colony. The diameter of the septate-hyphae is approximately 4 μm.

### 3.2. Mycelial Biomass Production

**Figure 3:**
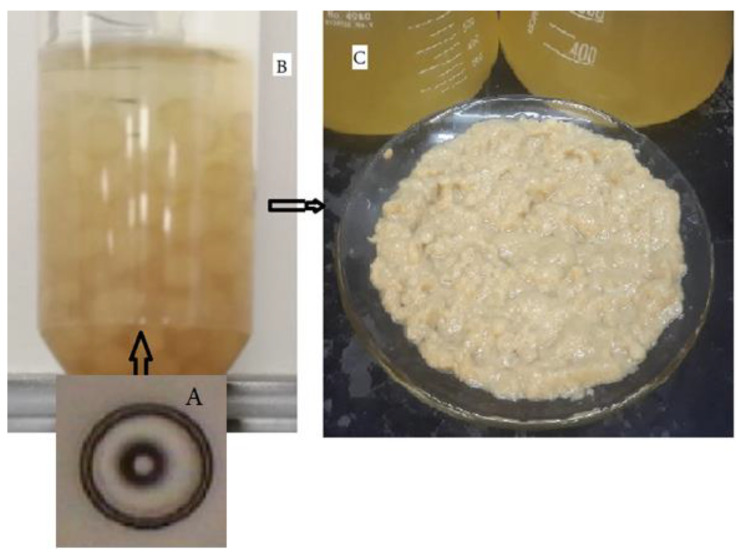
Mycelium and biomass produced on potato dextrose broth at 25 °C for two weeks using a shaking incubator at 200 rpm. (**A**) single ascospore of *T. nivea* (Spore diameter is equivalent to 16.5 μm), (**B**) type of mycelia produced on the growth medium, and (**C**) wet biomass.

The ascospores of *T. nivea* can be cultivated on potato dextrose broth to produce a spherical mass of mycelium (Figure 3A). The morphological characteristics of the biomass produced from laboratory cultivation are shown in Figure 3B. The yield (g of biomass/100 mL of broth) was 6 ± 1 (N = 3). 

### 3.3. GC–MS Analysis of Crud Extracts

The GC–MS analysis of crude methanol, ethyl acetate and hexane extracts obtained from the wet mycelial biomass was performed to screen the active biological compounds that could be produced from the mycelium. The results showed that the crude ethyl acetate, methanol and hexane extracts could be renewable resources of many biological compounds (Table 1, Table 2, Table 3, Table 4, Table 5 and Table 6). The results showed that the crude extracts are sources of many important compounds such as hexanal, dodecane, 4-hydroxyphenylacetic acid, heptadecane, 1-eicosene, hexa-decanoic acid-methyl ester, 7-pentadecyne, phenol, 2,4-bis(1,1-dimethylethyl), methyl palmitate, methyl linolelaidate, methyl oleate, methyl stearate, 9,12-Octadecadienoic acid (Z,Z)-, heptadecane, 1-octadecene, 5-hydroxymaltol, 11-dodecen-1-ol trifluoroacetate, and other compounds of medicinal and industrial importance listed in the tables. 

### 3.4. Biological Activity of Crude Alcoholic Extracts

The data from the disk diffusion test showed that all the extracts had biological activity against all the tested microorganisms. The inhibition zones resulting from the activity of the extracts ranged from 8 mm to about 13 mm. The findings showed that crude methanol hexane extracts of the mycelia-free broth had the highest biological activity against tested microbes among all extracts investigated in this work (Table 7).

## 4. Discussion

This study is the first of its kind to use the ascospores of *T. nivea* to produce mycelial biomass as a renewable resource of biological compounds. The yield of wet biomass and the compounds detected in fungal-cell free broth are encouraging for the consideration of industrial applications that might depend on this source. The GC–MS analysis of the mycelial biomass and by-products biosynthesized as extracellular compounds in a growth medium (potato dextrose broth) reported that mycelial biomass and its by-products are natural renewable resources of several important biological compounds that have many applications in the medical and industrial fields. The production of biological compounds from the fruiting body of *T. nivea* requires a long period of time and is affected by climate change, unlike the current method, in which all production conditions are controlled inside a laboratory.

The preliminary screening of chemical compounds of the crude ethyl acetate of wet mycelia biomass showed that the extract contained the following important biological compounds: hexanal (hexanaldehyde), which is applied in the flavor industry [16]; anthracene, which is used to produce dyes [17]; 8-methyloctahydrocoumarin, which can be used as a coumarin derivative [18]; dodecane, which is used as a solvent and a scintillator [19]; phenol, 2, 4-bis (1,1-dimethylethyl), which has many applications in medication and food [20]; 4-hydroxyphenylacetic acid, ethyl ester and tert-butyl-dimethyl-silyl, which is a 4-hydroxyphenylacetic acid derivative that plays an important role in metabolic reactions in plant, fungi, animals and humans[21]; heptadecane, which is used in the production of essential oils [22]; oleic acid, which has many applications in human and animal food as emulsifying agent and in cosmetics [23]; 1-eicosene, which is used as a monomer to produce several chemical copolymers [24]; hexa-decanoic acid methyl ester, which is an anticancer compound [25]; tetra-decanoic acid (i.e., myristic acid used in cosmetics) [26]; 9,12-octadecadienoic acid and methyl ester (linoleic acid), which is used as a hypocholesterolemic, anticancer and anti-inflammatory agent, among others [27]; and 7-pentadecyne, which is an anticancer compound [25].

Similar biological compounds were extracted using ethyl acetate from both wet mycelia biomass and mycelia-free broth, such as phenol, 2, 4-bis (1,1-dimethylethyl), heptadecane, hexa-decanoic acid methyl ester (which was also found in the crude methanol extract and crude hexane extract of wet mycelia biomass) and 1-eicosene. All the biological compounds listed in Table 2 were detected only in the crude ethyl acetate of wet mycelia biomass, except for the compounds mentioned above and cis-9-octadecenamide (i.e., oleamide, a drug for anxiety disorders) [28], which was also found in the crude hexane extract of wet mycelial biomass. The compounds presented in Table 2 include those that could be used in industrial applications, such as octadecanamide (stearamide) [29,30], stigmasta derivatives [31], 1-octadecene (which has an important role in nanoelectronics production) [32] and methyl decanoate [33].

Table 3 presents the following compounds, also found in other extracts: dodecane (found in crude ethyl acetate), 7-pentadecyne (found in crude ethyl acetate), hexa-decanoic acid methyl ester (found in the crude extract of ethyl acetate and crude hexane extract of wet mycelial biomass), 9,12-octadecadienoic acid (Z,Z)-methyl ester (found in crude ethyl acetate and hexane extracts of wet mycelial biomass), 9-octadecenoic acid (Z)-methyl ester (found in crude ethyl acetate and hexane extracts of wet mycelial biomass), methyl 16-methyl-heptadecanoate (found in the crude hexane extract of mycelium-free medium) and n-hexa-decanoic acid (found in the crude methanol extract of wet mycelial biomass and considered an anti-inflammatory agent [34] and a potential anticancer agent [35].

Regarding all the extracts screened in this work, the results showed that the crude methanol extract of the mycelium-free medium was the only resource of all the biological compounds listed in Table 4, compared with the other crude extracts analyzed in this work. The compounds obtained from the crude methanol extract of the mycelium-free medium have the following biological and industrial applications: antimicrobial and antiviral activities (e.g., 1-Piperidineethanol and its derivatives [36]glutaraldehyde [37], nonanoic acid [38] and dodemorph [39], perfume and flavor industries (e.g., 2,4-dihydroxy-2,5-dimethyl-3(2H)-furan-3-one [40] and maltol [41], solvent industry (e.g., 1,3-dimethyl-2-imidazolidinone [42], bio-refinery (e.g., methyl 2-furoate [43] and food and public health (e.g., 2-furancarboxaldehyde, 5-(hydroxymethyl) ([44].

The crude hexane extract of wet mycelial biomass (Table 5) was distinguished from the other crude extracts through the presence of the following compounds: 1,5-dimethyl-4-allylaminocytosine, 2(1H)-quinolinone, 4-phenoxy-, tri-tetracontane, carbonic acid, octadecyl 2,2,2-trichloroethyl ester, phthalic acid, 3-(2-methoxyethyl)heptyl propyl ester, penta-decyl heptafluorobutyrate, cyclohexadecane, 1,2-diethyl-, 2H-1,3-benzimidazol-2-one, 5-amino-1,3-dihydro-, 3-buten-2-one, 4-(di-methylamino)-3-(methylamino)-, 11-dodecen-1-ol trifluoroacetate, 2-mmethyl-7-nonadecene, E-15-heptadecenal, cyclohexane, 2-propenyl-, estra-1,3,5(10)-trien-17.beta.-ol, cyclohexene, 4-(4-ethylcyclohexyl)-1-pentyl, 1,2-benzenedicarboxylic acid and mono(2-ethylhexyl) ester. Benzimidazole and its derivatives are good examples of the important biological compounds of this extract that can be used in the biochemical synthesis of many antiparasitic [45] and antifungal agents [46].

As shown in Table 6, five compounds found in the crude hexane extract of mycelium-free medium were also found in the crude extracts of hexane (7,9-Di-tert-butyl-1-oxaspiro (4,5) deca-6,9-diene-2,8-dione, p-Menth-8(10)-en-9-ol, cis-, cyclohexane, 1-(1,5-dimethylhexyl)-4-(4-methylpentyl), pyridine-3-carboxamide, oxime and N-(2-trifluoromethylphenyl) acid] and methanol (methyl 16-methyl-heptadecanoate)). The crude hexane extract obtained from the mycelium-free medium included many important biological compounds, such as benzene-dicarboxylic acid and its derivatives, which play a role in the perfume and cosmetic industries [27]; phenylacetaldehyde derivatives, which are used in the aroma and flavor industries and have antimicrobial activity [47]; caryophyllenes, which have biological activities as antimicrobial, antioxidant and anti-inflammatory agents [48,49] phthalic acid esters and dibutyl phthalate, which can be used in many products such as plasticizers [50]; pentadecanoic acid, which has biological activity as an anticancer agent [51] ; and cyclo-pentadecane compounds, which have anti-trypanosomal activity for Chagas disease [52].

In industrial mycology, there are two major two forms of applied production, the first from fungal biomass (fungal cells) and the second from metabolic products which are frequently extracted from the cell-fee broth. In the present work, the findings reported significant variety between the compounds detected in mycelia-free broths and fungal biomass for all extracts used. For example, octa-decanamide (which has several industrial applications [30] was extracted using ethyl acetate from fungal cell-free broth but not from fungal cell biomass. The same applies to carbamic acid (whose esters are commonly used as insecticides [53] and its derivates. With respect to crude methanol extracts, for example, the dodemorph (fungicide agent [54] can be produced from the fungal cell-free broth using methanol in the primary extraction stage. Among all extracts obtained in this works, the crude methanol extract obtained from the mycelia-free broth was the only resource of several compounds such as dodemorph, 1-piperidineethanol, and maltol. The same result was repeated with regard to crude hexane extracts, where it was confirmed that fungal cell-free broth is considered a resource of many compounds that are absent from the fungal biomass. For example, caryophyllene, heptacosane, Tetratriacontane, dibutyl phthalate, and docosene.

Thomas et al., [55] reported that there are many industrial applications of Truffles including *T. nivea*. The applications include food, medicinal, and nanoparticles. In general, truffles (for example, *T. nivea*) are considered as one the most important sources of antioxidant agents such as phenolics, β-carotene, and ascorbic acid [56]. There are several antimicrobial agents detected in this work such as 1-Piperidineethanol and its derivatives (in the crude methanol extract of mycelia-free medium), Benzimidazole (in crude hexane extract of mycelial biomass and its derivatives), and dodemorph (in crude methanol extracts of mycelia-free medium). Some studies have reported that the fruiting body of *T. nivea* contains many antimicrobial agents [7,11] but there no work investigated the biomass produced from ascospores *T. nivea* as a source of potential antimicrobial agents.

## 5. Conclusions

This study investigated the preliminary chemical screening of the major volatile organic compounds of mycelia biomass produced from the ascospores *T. nivea* and metabolites secreted as extracellular products. The yield of wet mycelium biomass reached about 6 gm per 100 mL of medium; furthermore the fungal cell-free broth was the source of many important compounds. The findings show that the crude extracts of ethyl acetate, methanol and hexane obtained in this study could be natural renewable resources in several biological activity compounds that play the role of antibacterial, antifungal (for example, piperidine-ethanol, benzimidazole, and dodemorph), antiviral, anticancer, antioxidant, anti-trypanosomal and anti-inflammatory agents. The crude hexane and methanol of fungal cell-free broth extracts could be the best source of antimicrobial agents among all tested extracts. In addition, many compounds were found in all the crude extracts that could be used in industrial applications. We suggest confirming the benefits of this novel and renewable resource through small-scale production of its products and further investigation of its biological activity.

## Figures and Tables

**Table 1:** The GC-MS analysis of the crude ethyl acetate obtained from wet mycelial biomass produced using ascospores of *T*. *nivea*.

Compound Name	Chemical Formula	Molecular Weight (g/mol)	RT (min)	Quality	Area%
Hexanal	C_6_H_12_O	100.16	4.346	47	1
1H-Indene, 1-ethylideneoctahydro-7a-methyl-, cis-	C_12_H_20_	164.29	5.072	64	5.4
9-Nitroanthracene	C_14_H_9_NO_2_	223.23	7.756	9	0.8
3-(4-nitrophenyl)-1-phenylprop-2-en-1-one	C_15_H_11_NO_3_	253.25	7.96	47	1.9
8-Methyloctahydrocoumarin	C_10_H_16_O_2_	168.23	9.652	27	0.8
Dodecane	C_12_H_26_	170.33	10.212	49	2.7
(1E)-1-(4-Hydroxyphenyl)ethenone ((2Z)-3-methyl-1,3-benzothiazol-2(3H)xylidine)hydrazone	C_16_H_15_N_3_OS	297.4	13.081	22	1
5,9-Undecadien-1-yne, 6,10-dimethyl-	C_13_H_20_	176.3	13.648	35	1
2,4-Di-tert-butylphenol	C_14_H_22_O	206.32	15.035	76	1.4
beta-Cadinene	C_15_H_24_	204.35	15.162	46	0.9
4-Hydroxyphenylacetic acid, ethyl ester, tert-butyl-dimethyl-silyl	C_16_H_26_O_3_Si	294.46	15.569	38	2.5
1-Octadecanesulphonyl chloride	C_18_H_37_ClO_2_S	353	16.052	52	1.5
Heptadecane	C_17_H_36_	240.5	17.331	90	1.9
Nafoxidine	C_29_H_31_NO_2_	425.6	17.662	27	2.3
cis-7, cis-11-Hexadecadien-1-yl acetate	C_18_H_32_O_2_	280.4	18.502	55	1.9
7-Hexadecenal, (Z)-	C_16_H_30_O	238.41	18.61	53	1
Oleic Acid	C_18_H_34_O_2_	282.5	19.367	25	1.1
1-Eicosene	C_20_H_40_	280.5	19.59	83	1.6
Methyl palmitate	C_17_H_34_O_2_	270.5	19.889	98	11.5
Z,E-7,11-Hexadecadien-1-yl acetate	C_18_H_32_O_2_	280.4	19.952	72	1
Tetra-decanoic acid	C_14_H_28_O_2_	228.37	20.379	74	17.2
Phenol, o-(2-butenylsulfinyl)-	C_10_H_12_O_2_S	196.27	20.614	35	6
Pyrimidin-4-one, hexahydro-3-hydroxy-2-(4-nitrophenyl)-	C_10_H_11_N_3_O_4_	237.21	20.996	50	1.9
Methyl linolelaidate	C_19_H_34_O_2_	294.5	21.575	99	9.5
Methyl oleate	C19H36O2	296.5	21.625	99	10.9
Methyl stearate	C_19_H_38_O_2_	298.5	21.855	89	3
9,12-Octadecadienoic acid (Z,Z)-	C_18_H_32_O_2_	280.4	22.109	99	3.5
Z,Z-11,13-Hexadecadien-1-ol	C_16_H_30_O	238.41	22.408	56	2.2
7-Penta-decyne	C_15_H_28_	208.38	22.713	92	1.5
Pyrimidin-4-one, hexahydro-3-hydroxy-2-(4-nitrophenyl)-	C_10_H_11_N_3_O_4_	237.21	23.7	27	1.2

**Table 2:** The GC-MS analysis of the crude ethyl acetate obtained from mycelia-free medium that used to cultivation of ascospores of *T*. *nivea*.

Compound Name	Chemical Formula	Molecular Weight (g/mol)	RT (min)	Quality	Area%
2,4-Di-tert-butylphenol	C_14_H_22_O	206.32	15.009	97	59.3
4-Amino-7-diethylamino-chromen-2-one	C_13_H_16_N_2_O_2_	232.28	16.568	64	0.5
Heptadecane	C_17_H_36_	240.5	17.312	90	0.6
Methyl 8-methyl-decanoate	C_12_H_24_O_2_	200.32	17.649	42	0.7
3,5-di-tert-Butyl-4-hydroxybenzaldehyde	C_15_H_22_O_2_	234.33	18.292	95	0.4
1-Octadecene	C_18_H_36_	252.5	18.425	98	1.4
Octadecane	C_18_H_38_	245.5	18.495	86	0.4
2-Ethyl-1,3,4-trimethyl-3-pyrazolin-5-one	C_8_H_14_N_2_O	154.21	18.973	46	1
9-Methylnonadecane	C_20_H_42_	282.5	19.59	76	0.5
METHYL PALMITATE	C_17_H_34_O_2_	270.5	19.882	95	0.4
2,5-Cyclohexadien-1-one, 2,6-bis(1,1-dimethylethyl)-4-ethylidene-	C_16_H_24_O	232.36	19.965	78	4.4
Benzene, 1-methoxy-2-(methylthio)-	C_8_H_10_OS	154.229	20.175	43	1.2
BUTYL ISOBUTYL PHTHALATE	C_16_H_22_O_4_	278.34	20.353	80	1.4
1-Eicosene	C_20_H_40_	280.5	20.557	95	0.7
4-(2,2-dimethylpropionylamino)benzoic acid	C_12_H_15_NO_3_	221.5	20.601	15	0.9
4-(2-phenylquinazolin-4-yl)morpholine	C_18_H_17_N_3_O	291.3	20.773	93	1.5
Chlorpropham	C_10_H_12_C_l_NO_2_	213.66	21.365	52	1.5
Z-8-Hexadecene	C_16_H_32_	242.42	21.479	99	5.7
Cyclopentadecanone, 2-hydroxy-	C_15_H_28_O	240.38	21.625	93	0.5
3,4-Octadiene, 7-methyl-	C_9_H_16_	142.22	22.306	70	0.6
Octa-decanamide	C_18_H_37_NO	283.5	22.44	78	0.8
D-Homo-androstane (5.alpha.,13.alpha.)-	C20H_34_	274.5	23.241	84	0.3
2-(Diphenyl-phosphoryl)-4-nitrophenol	C_18_H_14_NO_4_P	339.3	23.973	90	1.2
9-Octadecenamide, (Z)-	C_18_H_35_NO	281.5	24.107	97	12.6
Stigmasta-4,6,22-trien-3.beta.-ol	C_29_H_46_O	410.7	25.265	93	0.4
2-Pyridinecarbohydrazonamide, N′-[(2,4-dimethoxyphenyl)methylidene]-	C_15_H_16_N_4_O_2_	284.31	25.468	95	0.7
Androsta[17-16-b]furan-5′-imine, 4′-methylene-3-methoxy-N-cyclohexyl-	C_29_H_45_NO_2_	439.7	26.238	42	0.5

**Table 3:** The GC-MS analysis of the crude methanol extract obtained from wet mycelial biomass produced using ascospores of *T*. *nivea*.

Compound Name	Chemical Formula	Molecular Weight (g/mol)	RT (min)	Quality	Area%
Dodecane	C_12_H_26_	170.33	10.231	53	4.4
7-Pentadecyne	C_15_H_28_	208.33	19.685	90	1.4
Methyl palmitate	C_17_H_34_O_2_	270.5	19.889	98	16.9
Palmitic acid	C_16_H_32_O_2_	256.42	20.353	90	10.4
Methyl linolelaidate	C_19_H_34_O_2_	294.5	21.575	99	18
Methyl oleate	C_19_H_36_O_2_	296.5	21.626	99	30
Methyl isostearate	C_19_H_38_O_2_	298.5	21.848	95	3.2
2,5-Dihydroxy-2-(4-methyl-pent-3-enyl)-2,3-dihydrobenzofuran-3,4-dicarboxylic acid, 3-ethyl ester 4-methyl ester	C_19_H_24_O_7_	364.4	27.491	27	5.2
1-[(Z)-3-Hydroxy-3-phenyl-1-triazenyl]anthra-9,10-quinone	C_20_H_13_N_3_O_3_	343.3	28.707	59	10.6

**Table 4:** The GC-MS analysis of the crude methanol extract obtained from mycelia-free medium that used to cultivation of ascospores of *T*. *nivea*.

Compound Name	Chemical Formula	Molecular Weight (g/mol)	RT (min)	Quality	Area%
1-Piperidineethanol	C_7_H_15_NO	129.2	6.331	72	1.6
5-Methyl furfural	C_6_H_6_O2	110.11	6.745	90	2.3
2,4-Dihydroxy-2,5-dimethyl-3(2H)-furanone	C_6_H_8_O_4_	144.12	7.025	52	0.8
1,3-Dioxane-2-propanol, 2-methyl-	C_8_H_16_O_3_	160.21	7.4	22	0.6
2-Hydroxy-gamma-butyrolactone	C_4_H_6_O_3_	102.09	7.572	53	3.2
trans-1,2,5,5-Tetramethyl-3,7,9-trioxabicyclo(4,2,1)nonane	C_10_H_18_O_3_	186.25	8.03	43	0.4
1,3-Dimethyl-2-imidazolidinone	C_5_H_10_N_2_O	114.15	8.253	47	0.8
2-Methyl-5-(methylthio)furan	C_6_H_8_OS	128.19	8.431	59	2.1
2-Methoxy-6-methylpyrazine	C_6_H_8_N_2_O	124.14	8.59	72	0.9
Methyl 2-furoate	C_6_H_6_O_3_	126.11	8.685	30	1.4
Glutaraldehyde	C_5_H_8_O_2_	100.12	8.914	50	3.6
Maltol	C_6_H_6_O_3_	126.11	9.137	83	0.6
4H-Pyran-4-one, 2,3-dihydro-3,5-dihydroxy-6-methyl-	C_6_H_8_O_4_	144.12	9.722	62	15.6
5-Hydroxymaltol	C_6_H_6_O_4_	142.11	10.346	91	0.6
5-hydroxymethe high-furfural	C_6_H_6_O_3_	126.11	11.109	91	38.9
5-Isopropenyl-2-methylpyridine	C_9_H_11_N	133.19	13.037	46	1.7
3-Furanacetic acid, 4-hexyl-2,5-dihydro-2,5-dioxo-	C_12_H_16_O_5_	240.25	14.589	74	21.6
Nonanoic acid	C_9_H_18_O_2_	158.24	15.308	55	1.6
2,3-Dimethyl-8-oxo-non-2-enal	C11H_18_O_2_	182.26	17.999	27	0.9
Dodemorph	C_18_H_35_NO	281.5	20.328	25	0.8

**Table 5:** The GC-MS analysis of the crude hexane extract obtained from wet mycelial biomass produced using ascospores of *T*. *nivea*.

Compound Name	Chemical Formula	Molecular Weight (g/mol)	RT (min)	Quality	Area%
1,5-Dimethyl-4-allylaminocytosine	C_9_H_13_N_3_O	179.22	5.065	38	2.3
3-(4-nitrophenyl)-1-phenylprop-2-en-1-one	C_15_H_11_NO_3_	253.25	7.954	47	0.8
4-Phenoxy-2-quinolinol	C_15_H_11_NO_2_	237.25	15.575	25	1.4
2,5-Cyclohexadien-1-one, 2,6-bis(1,1-dimethylethyl)-4-ethylidene-	C_16_H_24_O	232.36	16.568	62	0.7
Tritriacontane	C_43_H_88_	605.2	17.331	58	1.1
Carbonic acid, octadecyl 2,2,2-trichloroethyl ester	C_21_H_39_C_l3_O_3_	445.9	19.214	60	1.2
Phthalic acid, 3-(2-methoxyethyl)heptyl propyl ester	C_21_H_32_O_5_	364.5	19.367	59	0.7
Penta-decyl heptafluorobutyrate	C_19_H_31_F_7_O_2_	424.4	19.526	93	0.9
METHYL PALMITATE	C_17_H_34_O_2_	270.5	19.882	97	4.3
7,9-Di-tert-butyl-1-oxaspiro(4,5)deca-6,9-diene-2,8-dione	C_17_H_24_O_3_	276.4	19.946	97	5.6
Cyclo-hexadecane, 1,2-diethyl-	C_20_H_40_	280.5	20.181	70	0.7
5-Amino-1,3-dihydro-2H-benzimidazol-2-one	C_7_H_7_N_3_O	149.15	20.359	46	5.9
3-Buten-2-one, 4-(dimethyl-amino)-3-(methylamino)-	C_7_H_14_N_2_O	142.2	20.595	46	16.3
11-Dodecen-1-ol trifluoroacetate	C_14_H_23_F_3_O_2_	280.33	20.868	90	0.8
Pyrimidin-4-one, hexahydro-3-hydroxy-2-(4-nitrophenyl)-	C_10_H_11_N_3_O_4_	237.21	20.996	44	0.6
2-Methyl-7-nonadecene	C_20_H_40_	280.5	21.307	86	1
E-15-Heptadecenal	C_17_H_32_O	252.4	21.473	97	4.5
Methyl linoleate	C_19_H_34_O_2_	294.5	21.575	99	2
Methyl oleate	C_19_H_36_O_2_	296.5	21.626	99	7.5
Methyl stearate	C_19_H_38_O_2_	298.5	21.848	86	1.6
Linolic acid	C_18_H_32_O_2_	280.4	22.128	70	1.2
Allyl-cyclo-hexane	C_9_H_16_	124.22	22.332	62	1.3
Pyridine-3-carboxamide, oxime, N-(2-trifluoromethylphenyl)-	C_13_H_10_F_3_N_3_O	281.23	22.402	92	0.7
3-Deoxy-17.beta.-estradiol	C_18_H_24_O	256.4	22.586	49	0.8
Cyclohexene, 4-(4-ethylcyclohexyl)-1-pentyl-	C_19_H_34_	262.5	22.707	56	1.4
p-Menth-8(10)-en-9-ol	C_10_H_18_O	154.25	22.796	53	0.6
Cyclohexane, 1-(1,5-dimethylhexyl)-4-(4-methylpentyl)-	C_20_H_40_	280.5	23.178	92	1.1
9-Octadecenamide, (Z)-	C_18_H_35_NO	281.5	24.145	93	30.7
Pyridine-3-carboxamide, oxime, N-(2-trifluoromethylphenyl)-	C_13_H_10_F_3_N_3_O	281.23	24.883	95	1
1,2-Benzenedicarboxylic acid, mono(2-ethylhexyl) ester	C_16_H_22_O_4_	278.34	25.468	52	1.7

**Table 6:** GC-MS analysis of the crude hexane extract obtained from mycelia-free medium used in cultivation of ascospores of *T*. *nivea*.

Compound Name	Chemical Formula	Molecular Weight (g/mol)	RT (min)	Quality	Area%
2-(2-Methoxy-5-methyl-phenyl)-propionaldehyde	C_11_H_14_O_2_	178.23	5.091	35	0.9
Viridicatol	C_15_H_11_NO_3_	253.25	7.96	38	0.7
Heptacosane	C_27_H_56_	380.7	10.238	58	1
Caryophyllene	C_15_H_24_	204.35	13.648	58	0.7
Naphthalene, 1,2,3,5,6,8a-hexahydro-4,7-dimethyl-1-(1-methylethyl)-, (1S-cis)-	C_15_H_24_	204.35	15.162	83	0.6
Tetratriacontane	C_34_H_70_	478.9	17.325	72	0.7
alpha.-Bisabol oxide B	C_15_H_26_O_2_	238.37	17.662	35	2.5
5-Dimethylamino-furan-2-carbaldehyde	C_7_H_9_NO_2_	139.15	19.202	43	4.7
Phthalic acid, isobutyl non-5-yn-3-yl ester	C_21_H_28_O_4_	344.4	19.367	72	1.8
Methyl 14-methylpentadecanoate	C_17_H_34_O_2_	270.5	19.882	97	3
7,9-Di-tert-butyl-1-oxaspiro(4,5)deca-6,9-diene-2,8-dione	C_17_H_24_O_3_	276.4	19.946	93	2
9,12-Octadecadienoic acid (Z,Z)-	C_18_H_32_O_2_	280.4	20.124	44	1.1
Dibutyl phthalate	C_16_H_22_O_4_	278.34	20.366	81	13.1
p-Menth-8(10)-en-9-ol	C_10_H_18_O	154.25	20.875	90	0.8
3-Eicosene, (E)-	C_20_H_40_	280.5	21.307	89	0.7
n-Nonadecanol-1	C_19_H_40_O	284.5	21.467	95	2.8
Methyl vaccenate	C_19_H_36_O_2_	296.5	21.626	99	3.2
Methyl 16-methyl-heptadecanoate	C_19_H_38_O_2_	298.5	21.848	96	2.1
(1-Propylnonyl)cyclohexane	C_18_H_36_	252.5	22.332	64	0.9
1-Docosene	C_22_H_44_	308.6	22.44	98	2.8
Cyclo-pentadecane	C_15_H_30_	210.4	22.586	90	1.3
Cyclohexane, 1-(1,5-dimethylhexyl)-4-(4-methylpentyl)-	C_20_H_40_	280.5	23.184	94	0.7
Bicyclo[2.1.1]hexane-1-carboxylic acid, 5,5-dimethyl-	C_9_H_14_O_2_	154.21	23.413	87	0.9
Bis(2-ethylhexyl) adipate	C_22_H_42_O_4_	370.6	24.234	95	12.1
Dicyclo-hexyl phthalate	C_20_H_26_O_4_	330.4	25.366	87	9.9
Diiso-octyl phthalate	C_24_H_38_O_4_	390.6	25.468	86	15.4
Pyridine-3-carboxamide, oxime, N-(2-trifluoromethylphenyl)-	C_13_H_10_F_3_N_3_O	281.23	25.748	93	2.2
Isophthalic acid, di(4-octyl) ester	C_24_H_38_O_4_	390.6	26.938	68	11.3

**Table 7:** The biological activity of the crude extracts obtained from the biomass of *T. nevia* and mycelia-free medium (N = 3).

		Inhibition Zone (mm) ± Std. Deviation		
		N	Mean	Std. Deviation
*E. coli* ATCC 25922	Crude ethyl acetate of biomass	3	9.3	0.57735
	Crude ethyl acetate of mycelia-free medium	3	10	0
	Crude methanol extract of biomass	3	8.6	0.57735
	Crude methanol extract of mycelia-free medium	3	11.3 *	0.57735
	Crude hexane extract of biomass	3	8.6	0.57735
	Crude hexane extract of mycelia-free medium	3	11.6 *	0.57735
	Total	18	9.9	1.30484
*S. aureus* ATCC 29213	Crude ethyl acetate of biomass	3	9.3	1.1547
	Crude ethyl acetate of mycelia-free medium	3	9.6	1.1547
	Crude methanol extract of biomass	3	9.3	0.57
	Crude methanol extract of mycelia-free medium	3	11.0 *	0
	Crude hexane extract of biomass	3	8.3	0.57
	Crude hexane extract of mycelia-free medium	3	12.0 *	1
	Total	18	9.9	1.43
*C. albicans* ATCC 60193	Crude ethyl acetate of biomass	3	10.6	0.57
	Crude ethyl acetate of mycelia-free medium	3	11.0 *	1
	Crude methanol extract of biomass	3	10.3	1.15
	Crude methanol extract of mycelia-free medium	3	13.0 *	0
	Crude hexane extract of biomass	3	10	1
	Crude hexane extract of mycelia-free medium	3	12.6 *	0.57
	Total	18	11.2	1.36
*A. niger* Wild strain	Crude ethyl acetate of biomass	3	8.3	0.57
	Crude ethyl acetate of mycelia-free medium	3	9.3	0.57
	Crude methanol extract of biomass	3	8.6	0.57
	Crude methanol extract of mycelia-free medium	3	9	0
	Crude hexane extract of biomass	3	9	1
	Crude hexane extract of mycelia-free medium	3	8.3	0.57
	Total	18	8.7	0.64

* The means are significantly different (*p* < 0.05) using One-Way ANOVA: Post Hoc multiple comparisons, Tukey test (IBM SPSS, Statistics 25).

## Data Availability

Not applicable.
